# Addressing youths’ climate change-related distress: a qualitative study on the experience of burden, triggering and protective factors

**DOI:** 10.1136/bmjment-2025-301549

**Published:** 2025-10-02

**Authors:** Henrik Wasmus, Leonie Fleck, Tim Schmidt, Stefan Scheydt, Frederike Schirmbeck, Evaldas Kazlauskas, Wietse Tol, Ulrich Reininghaus

**Affiliations:** 1 Public Mental Health, Central Institute of Mental Health, Mannheim, BW, Germany; 2 Gesundheit, Berner Fachhochschule, Bern, BE, Switzerland; 3 Center for Psychotraumatology, Institute of Psychology, Vilnius University, Vilnius, Vilnius County, Lithuania; 4 Department of Public Health, University of Copenhagen, Copenhagen, Capital Region of Denmark, Denmark; 5 Athena Research Institute, Vrije Universiteit Amsterdam, Amsterdam, The Netherlands; 6 German Center for Mental Health (DZPG), partner site Mannheim-Heidelberg-Ulm, Mannheim, BW, Germany

**Keywords:** Child & adolescent psychiatry, Mental Health, Stress Disorders, Traumatic

## Abstract

**Background and objective:**

In recent years, growing scientific and public awareness has highlighted the negative impacts of climate change on mental health, particularly among young people, who are disproportionately affected. These findings underscore the need for effective and scalable interventions to support individuals experiencing climate change-related distress (CCD). At the initial stage, it is crucial to understand how this distress manifests and what the momentary risk and protective factors are that exacerbate and modulate its dynamic occurrence in everyday life.

**Methods:**

In this context of need, nine qualitative, semistructured interviews with young individuals, aged between 14 and 25 and living in Germany, with CCD were conducted. Interviews centred on individuals’ burdens, putative triggers eliciting the experience, as well as putative protective factors. We analysed the data and developed themes via Braun and Clarke’s reflexive thematic analysis and electively structured the analysis according to the coding paradigm adopted from Strauss and Corbin.

**Results:**

Participants reported experiencing a wide range of negative emotions as well as mental health difficulties associated with climate change, including sleep disturbances, reduced well-being and difficulties concentrating. The experience emerges from the understanding and awareness of the complexity of climate change and its associated consequences for the environment. Protective factors were reported, including positive emotions (eg, hope, finding meaning and purpose), self-efficacy, conceptual knowledge about climate change-related emotions and external factors (ie, social support). Participants employed various strategies to regulate their emotions, ranging from avoidance and distraction to strategies like acceptance, cognitive reappraisal and active engagement in pro-environmental behaviour or activism.

**Conclusion and clinical implications:**

Overall, this study enhances our understanding of young individuals’ emergence and daily life experience of CCD. The findings suggest that a prolonged or overly extensive occurrence may result in mental health difficulties. Moreover, the results highlight the importance of strengthening factors associated with resilience at a young age, enabling individuals to cope with CCD. The findings have implications for the development of potential intervention components and suggest imparting conceptual knowledge and adaptive regulatory strategies, supporting habit formation and providing networking opportunities with others affected by CCD.

**WHAT IS ALREADY KNOWN ON THIS TOPIC**

- Climate change is associated with youth mental health, ranging from negative emotions to functional impairment.
- In order to address climate change-related distress (CCD), it is important to understand its development, along with putative risk and protective factors.

**WHAT THIS STUDY ADDS**

- Our qualitative study provides in-depth insights into young people’s experience of CCD, detailing their perspective on the conditions and strategies which may modulate their everyday occurrence and consequences.

**HOW THIS STUDY MIGHT AFFECT RESEARCH, PRACTICE OR POLICY**

- The findings point towards helpful strategies for young people which may promote resilience in the context of climate change, including psychoeducation about climate change-related emotions, emotion regulation strategies, habit formation and opportunities to connect with like-minded people, which can be implemented in prevention and intervention programmes.
- In addition, this study offers putative pathways to CCD that warrant further investigation in future quantitative research.

## Background

 Climate change is one of the most pressing issues facing society today, and^1^ recently, increasing scientific and public awareness has drawn attention to the negative impacts of climate change not only on physical but also on mental health.^1^ Mental health can be affected directly (eg, through extreme weather events), indirectly (eg, through social, economic and environmental consequences for society) and in anticipation, through increased attention and awareness of the consequences.^1 2^

The diverse impacts of climate change can manifest as a range of mental health difficulties.^3^ Moreover, findings from a large, representative survey of 10 000 young people (aged 16–25) suggest that, whereas only a small proportion of young people may be impacted by climate change in such a way to meet criteria for a clinical mental health condition, a substantial proportion experiences distress and negative emotional responses in relation to climate change.^4^ Although negative emotions in response to climate change have been reported to be associated with more pronounced mental health difficulties, these need not be pathological per se.^3 5^ Instead, they represent a natural response to an existential threat and have also been linked to adaptive behaviours, such as pro-environmental actions^6^ and activism.^7^ Consequently, authors in the field suggest drawing a distinction between climate change-related distress (CCD) and climate change-related impairment (CCI).^6^

A report by the American Psychological Association (APA) states that climate change is a destabilising force that may exacerbate pre-existing social and structural inequalities, that is, socioeconomic disadvantage, age or pre-existing physical or mental health conditions.^1^ Thus, particular groups may be more susceptible to experiencing mental health difficulties due to climate change and, hence, reflect a priority target group for mental health promotion and prevention strategies. A recent scoping review suggests that maladaptive behaviour and mental health symptoms are more likely to emerge from CCD in individuals who directly experience the impacts of climate change, lack effective coping strategies or social support, or perceive insufficient attention from the government.^8^ Moreover, younger people appear to represent a subgroup particularly affected by CCD.^9^ There is evidence that awareness and greater susceptibility in young people is due to the more widespread acknowledgement of climate change in school curricula, news media and social media.^10^ Evidence further proposes an explanation by stating that younger people also tend to be more limited in adequate psychological coping strategies for dealing with threats appropriately.^11^ Yet, there has not been sufficient research about how young people experience CCD and CCI.

In the light of these findings, the need for the development of effective, cost-effective and scalable interventions that support individuals who experience CCD and CCI has been emphasised in a recent position paper of the APA.^12^ To achieve this, it is essential to understand how CCD and CCI manifest, as well as the momentary risk and protective factors that influence their occurrence and intensity.^1^ Individual resilience factors (eg, higher levels of self-efficacy and expressive flexibility) have been deemed to be important factors that may modulate CCD and CCI experience.^13^ Moreover, coping strategies (especially emotion regulation strategy of meaning-focused coping, which enables individuals to find meaning and reorient themselves towards positive goals) have been found to be associated with more positive affect, life satisfaction and optimism in relation to climate change.^14^ A conceptual paper further enquires about the importance of strengthening individuals’ problem-solving abilities as a longer-term strategy to prevent CCI.^15^ However, overall, there is still a lack of empirical data on factors that modulate CCD and CCI experience.^1^ What is more, significant gaps between scientific evidence on effective interventions and their implementation in real-world settings exist.^16 17^ Hence, understanding more fully which protective and triggering factors individuals with CCD and CCI identify as relevant to their experience, and taking their perspectives and insights into account is important in the design, evaluation and implementation of mental health promotion and prevention strategies for this priority target population.

## Objective

In this qualitative study, we aimed to explore young people’s subjective views and experience of CCD and CCI, as well as putative risk and protective factors. Specifically, we aimed to address the following research questions:

1. How do CCD and CCI occur and in which ways do they appear in the daily lives of young individuals?
2. What are general and momentary putative risk factors eliciting the experience of CCD and CCI?
3. What putative protective factors and regulating strategies, hindering the occurrence or regulating existing CCD, do young individuals possess in general and in daily life?

## Methods

The study was conducted in accordance with the Consolidated Criteria for Reporting Qualitative Research (COREQ) checklist^18^ and received ethical approval from the local ethics committee of the Mannheim Medical Faculty of the University of Heidelberg (2022-550), in line with the Declaration of Helsinki.^19^ All participants provided written informed consent, with parental consent additionally obtained for those under 18. Inclusion criteria included age 14–25 years, language proficiency in German, self-reported CCD.

Semistructured, guideline-based individual interviews were conducted either in person or online via a secured video platform (RED Connect; RED Medical Systems GmbH), depending on the participant’s preference. The face-to-face interviews took place in designated rooms at the Center for Innovative Psychiatric and Psychotherapeutic Research (CIPP), Central Institute of Mental Health in Mannheim. The first author (HW), a male PhD student and research associate with a background in clinical psychology, conducted all interviews individually with each of the participants. The interviewer had no prior personal relationship with the participants. Experience in qualitative research methods was gained through individual training and participation in a qualitative research colloquium. In addition, a theoretical understanding of the research topic was obtained before the study was conducted. The interview guide (see online supplemental file 1) was piloted and adapted via think-aloud methodology^20^ prior to the first interview with three independent individuals, who met inclusion criteria. Participants were recruited through local schools, universities and the member networks of a previously established study advisory group using purposive sampling.^21^ The criterion for stopping data collection was *theoretical saturation*. We analysed data iteratively, comparing codes and categories across all interviews. Saturation was reached at the point at which analysis of the data no longer provides new information or insights.^22^

The interviews were conducted in German, the native language of the interviewer and participants, between August and November 2024, lasting 40–60 min. Field notes documented key aspects and notable observations. Interviews were audio-recorded via a recording device and pseudonymised transcription was carried out using the AI-based software NoScribe (V.0.4.1),^23^ locally on a computer without internet access. The first author reviewed and corrected all transcripts by listening to the audio recordings again. Participants did not evaluate the transcripts. Coding of the transcripts was started within 2 days of each interview, using an inductive approach and following Braun and Clarke’s thematic analysis procedures^24^ using MAXQDA 2024 (V.24.6.0). The first three interviews were coded independently by the first author and coauthor TS (a PhD student with a background in implementation science). Coding systems were compared among coders until a consistent coding system was established and used for subsequent interviews. The final coding system was then discussed in a qualitative research colloquium, evaluated against the research objectives and refined into categories and themes. Participants were not contacted to provide feedback on categories and themes. To structure the resulted identified themes, the coding paradigm by Strauss and Corbin^25^ was electively applied. Here, the so-called central phenomenon is the focal point. Its causes, context and intervening conditions leading to the phenomenon, as well as resulting strategies and emerging consequences, are mapped. This paradigm was adapted and applied to the material with the intention of integrating the observed, isolated observations into a cohesive structural framework. Data were analysed in German and codes were subsequently translated into English.

## Findings

Nine individuals who met eligibility criteria were contacted and found willing to participate. All participants lived in Germany, in the Rhine-Neckar region. Table 1 displays the demographic characteristics of the participants.

**Table 1:** Demographic characteristics of the participants

ID	Age range	Gender	Current education level
**01**	20–25	Non-binary	University student
**02**	20–25	Female	University student
**03**	20–25	Female	University student
**04**	20–25	Female	University student
**05**	20–25	Male	University degree
**06**	20–25	Male	University student
**07**	14–19	Female	Secondary school
**08**	14–19	Male	Secondary school
**09**	14–19	Male	Secondary school

The age of participants is reported in a range for anonymity purposes.

**Figure 1:**
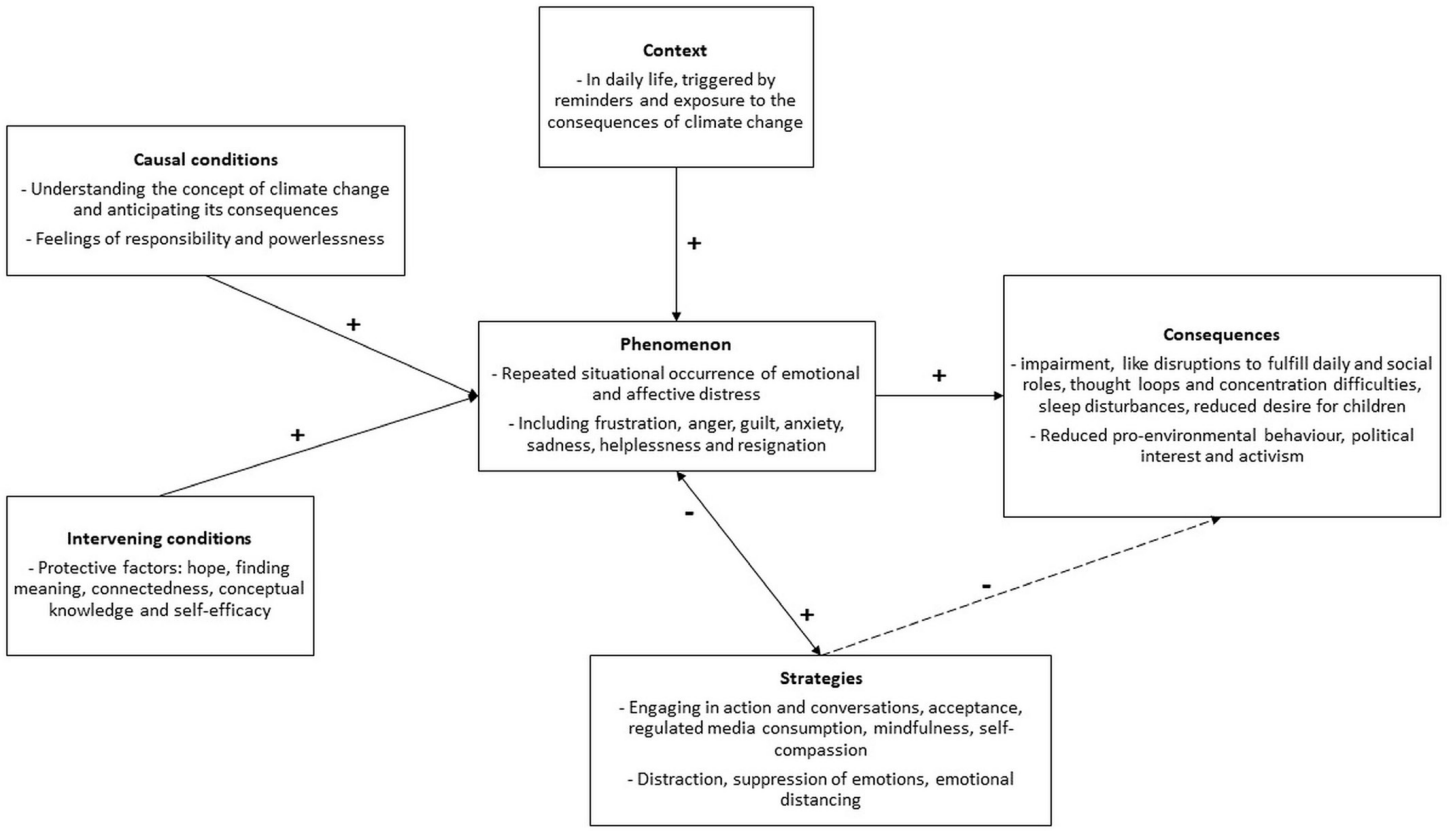
A model of climate change-related distress of young individuals, organised according to the coding paradigm described by Strauss and Corbin.^25^

The study results were organised according to the coding paradigm by Strauss and Corbin, as illustrated in figure 1.

In the coding paradigm, the *central phenomenon* refers to the conceptually defined and theorised occurrence under study. In this study, the central phenomenon of interest was the repeated situational occurrence of psychological distress related to climate change. Participants’ reported diverse negative emotions, including most commonly frustration, anger, guilt, anxiety, sadness, helplessness and resignation. Frustration and anger were predominantly directed towards economic and political systems prioritising interests over environmental protection. Some participants also expressed anger towards individuals in positions of power whose actions or inactions contributed to environmental damage.

If somehow a few hundred, a few thousand people who are at the very top, who own the companies, don’t change (…) there is a certain frustration. There are so many people who are committed to the climate, so many people who are betting their lives on climate research, and just a hundred people who are already rich without any limits, that out of pure greed they say, no, let’s not change, I don’t care what happens after I’m gone. (ID 05)

Anger also arose in response to the environmentally damaging behaviours by others. Anxiety was linked to concerns about the future. As one participant noted: “For this 1.5 degree goal there are only 9 years left (…) it’s getting closer and closer” (ID 09). Participants also highlighted fear of uncertainty regarding the scale and nature of upcoming changes, describing it as a “vast, yet undefined transformation” (ID 01). Sadness arose from witnessing the suffering of nature and the environment. One participant criticised: “When people have such a beautiful world, why do they just destroy it?” (ID 07). Feelings of guilt were commonly reported in the context of personal consumption choices and perceived inadequacies in climate activism. Almost all participants described experiencing helplessness, particularly in the face of perceived limitations of their political activism: “I’ve realised that my activism doesn’t have the reach I wish it had” (ID 01). For some, helplessness progressed to resignation. Additionally, many participants reported subtle distress, such as low mood, unease and negative physical sensations when thinking about climate change.

*Causal conditions* refer to factors that may elicit the *phenomenon*. Some participants described that their emotional response began with an understanding of climate change and its associated complexities, including the perception and anticipation of its environmental impacts. This involved recognising the catastrophic consequences of climate change if current trends continue, with a future-oriented focus: “When it really had this emotional effect was when I realized that this is a huge crisis and what exactly happens with a one-degree increase” (ID 05). Additionally, some participants described empathy and compassion towards nature, often accompanied by a sense of personal responsibility to act: “I think especially in the beginning, when the topic was still new to me, I thought I could and had to make a difference” (ID 03).

*Context* refers to specific conditions that may elicit the *phenomenon* in daily life. Many participants described emotional distress arising, “In moments when ie,ngaged with the consequences of climate change” (ID 05). Such moments were often prompted by reminders such as news reports focusing on problems and disasters, or by visible impacts of climate change, such as floods or droughts. These visible impacts were particularly relevant when they directly affected participants’ living environments, making the changes and future consequences more tangible: “… especially when I visit my parents, who live at the countryside. There, you can see everyday impact on nature and agriculture that have been caused by climate change and severe drought” (ID 01). Additionally, witnessing behaviours linked to the causes of climate change, such as others’ consumption choices or attitudes, was identified as a trigger (“What actually burdens me much more is what is more in front of my eyes. And that is this littering problem, which is directly human-made.” (ID 02)).

*Intervening conditions* refer to additional factors that may modulate the *phenomenon*, positively or negatively. Participants named protective factors that mitigate the occurrence or intensity of CCD. Positive emotions, such as hope and interest, emerged, alongside finding meaning and a sense of purpose, which fostered the belief that their actions could make a difference—particularly when acknowledged by others. Moreover, experiences of self-efficacy were reported: “I feel like I’m moving in a direction where I can actually make a difference” (ID 02), most prominently in situations connected to work, activism or interactions with peers. Participants further reported negative shifts in their sense of self-efficacy, especially when their limited impact of individual behaviour on climate change became salient or when they less frequently engaged in activism. Reduced self-efficacy seemed to be, in turn, associated with heightened emotional distress, often manifested as guilt (“I think I’m crying right now because I’ve somewhat lost my sense of self-efficacy, because I had such a narrow focus, studying for my exam, but you keep thinking to yourself, I really should be doing something” (ID 03)). A sense of connectedness, particularly within social networks of like-minded individuals, was reported as a key protective factor, fostering community and hope. Also, conceptual knowledge—about emotions and burdens associated with climate change—was reported to positively impact the *phenomenon*: “I think I can better identify what these emotions are, and then move past them more quickly” (ID 06).

*Strategies* describe actions taken by individuals to deal with the *phenomenon*. A recurring theme in this study was the tendency to take action, with participants emphasising that engaging in pro-environmental behaviour and activism helped them distract and regulate their distress.

My behaviour helps me because I can channel this emotionality, which is otherwise so unfocused, into actions. This makes me feel less powerless and helpless, and it allows me to reduce the negative emotions somewhat. (ID 04)

Some participants reported that acting in alignment with their values fostered self-efficacy: “it feels like a problem that can be solved, and as a result, like a task—this gives life a bit more meaning” (ID 02). Others described managing their emotions by developing an acceptance of their personal sphere of influence—clarifying what lay within their responsibility and what they could realistically achieve. Moreover, engaging in conversations with like-minded about the emotional distress was frequently mentioned as a way to process it. Other strategies named included (1) engaging in conversations with like-minded individuals, (2) regulated media consumption, (3) mindfulness and meditation, (4) positivity and (5) self-compassion.

Importantly, many participants reported using strategies involving emotional distancing. Some attempted to reduce feelings of guilt by rationalising their behaviour in comparison to others: (“One strategy is to think that others do the same.” ID 03). Others relied on suppression, avoidance or distraction to disengage from distressing emotions: “I think I’m currently very quick to push the thought away and distract myself” (ID 03).

*Consequences* refer to the direct consequences of the *phenomenon*, as well as the outcomes of the *strategies*. Participants described that prolonged or overly intense experience of CCD could lead to significant impairment, affecting mental health and daily functioning. One participant reflected: “In terms of current burdens, it strongly affects my work, my studies, and even daily life. (…) the climate catastrophe is such a topic that I find very difficult to talk about or deal with because I can’t really contain myself – I get overwhelmed by my feelings” (ID 01). Social strain was another common consequence, described as recurring conflicts with peers and strangers based on different views on climate change-related matters, in some cases resulting in distancing from former close personal relationships: “I’ve distanced myself from people, who are close to me and don’t feel as connected anymore. It’s obviously a burden when these are friends” (ID 04). Participants also reported persistent thoughts and mental imagery related to climate change, which led to concentration difficulties (“I was terribly distracted, living too much in my own head” ID 02) and repetitive thought loops (“I just make things worse in my head… I often have somewhat destructive thought streams” (ID 08). This was described by one participant as an ongoing mental occupation, resulting in reduced sleep quality (“I couldn’t sleep well, thinking about everything happening around us” ID 08). A notable consequence mentioned was a change in family planning due to concerns about climate impacts. As they explained: “It became a burden because I always thought I wanted kids. But then I questioned whether it’s even reasonable to bring children into a world where we might have malaria, Zika virus, or tiger mosquitoes here in Germany” (ID 05).

Some participants reported a decrease in burden over time, attributed to emotional distancing and desensitisation (“the everyday emotions have become less, so there is a certain desensitization, because this mental load is just too much” ID 04). Others noted a decline in pro-environmental behaviour and political engagement, attributing this to the responsibility of large corporations (“I think I became less engaged when I realized that big companies are responsible for this” ID 08). One participant linked this disengagement to resignation (“Well, it was something people were aware of, but (…) then I noticed more and more that people just don’t really care. For me, it increasingly became a ‘do whatever you want’ attitude.” ID 09).

## Discussion

This study moved beyond previous research by exploring subjective experiences of CCD and CCI, as well as general and daily life putative risk and protective factors in young people.

### Subjective experience of CCD and CCI in daily life

In line with existing literature,^1^ participants experienced repeated psychological distress due to climate change. Responses comprised multifaceted emotions linked to politics, society, others or personal behaviour and impact. Consistent with findings that CCD negatively affects daily life,^4^ participants reported significant functional and mental health impairment, including disruptions to occupational and social roles, concentration difficulties, heightened cognitive load and sleep disturbances. Extending previous research,^4^ this study provides initial insights into the subjective pathways from CCD to impairment. Reports suggest that overly frequent or intense CCD may result in, or exacerbate, impairment. Moreover, while CCD and related emotions are not inherently pathological and can motivate pro-environmental behaviour,^6 7^ our findings indicate that insufficient handling of CCD may reduce activism, political commitment and pro-environmental behaviour. These insights highlight the need to further examine both direct pathways from CCD to CCI and indirect pathways involving protective factors.

Participants described a wide range of emotion regulation strategies and protective factors which they experienced as helpful in mitigating negative consequences of CCD. This underscored the importance of equipping individuals with such strategies and fostering resilience from an early age in order to manage CCD effectively and prevent its progression to CCI. Measures of prevention and mental health promotion should aim to mitigate CCD’s negative impacts while enhancing its motivational potential for pro-environmental action. Ecological Momentary Assessment^26^ offers a promising method to capture real-time experiences of CCD and CCI, as well as the factors influencing these processes, in greater detail.

### Subjective experience of putative risk factors for CCD

Based on the coding paradigm by Strauss and Corbin,^25^ we identified putative subjective risk factors for CCD that may be grouped into more distal factors and proximal ones, that is, momentary triggers in daily life. Participants reported that initial burden stemmed from understanding the climate crisis’s complexity, including its effects on nature, environment and society. A key factor was recognising the limited impact of individual actions compared with companies, politicians and other high-level entities, consistent with reports in other studies linking CCD to dissatisfaction with governmental responses.^4^ This awareness fostered feelings of responsibility, paired with frustration and helplessness due to perceived limited self-efficacy. Aligned with the transactional model of stress and coping,^27^ participants viewed the climate crisis as a largely uncontrollable threat. Situational triggers included moments when being confronted with, reminded of, or reflecting on the crisis. News salience and visible catastrophic consequences were particularly relevant in eliciting CCD. These findings stress the role of how individuals learn about the climate crisis, especially their perception of personal influence. Media, schools and other stakeholders should present information carefully, as the mode of delivery strongly affects young people’s well-being. Consistent with existing guidelines,^28^ emphasising both individual influence and systemic accountability appears crucial for mitigating distress and fostering constructive engagement.

### Subjective experience of putative protective factors

Our findings suggest that protective factors and coping strategies for CCD can be grouped into conditions and strategies. Protective factors included positive emotions such as hope, finding meaning and purpose, self-efficacy, and conceptual knowledge about climate change-related emotions. Supporting existing research,^8^ external factors like social support from like-minded individuals and feelings of connectedness were reported as significant in reducing CCD. Future quantitative studies should examine these protective factors and their associations with CCD in more detail. Participants also described a range of emotion regulation strategies, aligning with existing distinctions between maladaptive and adaptive strategies.^29^ Reported maladaptive strategies (suppression, avoidance, distraction) may provide short-term relief but are linked to long-term mental health difficulties. By contrast, strategies such as acceptance, cognitive reappraisal and active engagement in behaviours were identified, referred to as adaptive strategies.^29^ Participants noted that CCD may result in CCI when they are unable to regulate CCD successfully. Considering these findings, the short-term and long-term impact of different strategies on mental health outcomes requires further scrutiny by future studies.^14^

A main limitation of this study is that qualitative data allow for multifaceted interpretations rather than a single one. The interview structure and guideline were developed, and data were analysed, primarily by the first author, who had theoretical understanding of the concepts under study. We undertook efforts to minimise researcher bias, including independent intercoding and discussions within a research colloquium, but cannot fully eliminate its potential impact. Furthermore, this study forms part of a doctoral thesis and aims to present an initial model of young people’s experiences with CCD. Future quantitative studies should validate this model by testing the proposed putative pathways. Additionally, the sample primarily included individuals with higher education and exclusively comprised individuals from Germany, a Western high-income country and less affected by climate crisis compared with other regions. Moreover, the entire sample consisted of young individuals who self-identified as being distressed by climate change, which limits the generalisability of the findings to a broader population. Participants were also not directly affected by severe weather or other significant events resulting from climate change. Hence, findings reflect putative indirect impacts of climate change on mental health and do not generalise to populations more directly affected by climate change. Future qualitative research should include perspectives of individuals directly affected by climate change, in accordance with theoretical saturation.

## Clinical implications

This study highlights the multifaceted psychological responses associated with climate change distress (CCD) among young individuals. The findings reveal that subjective experience of CCD originates from a deep understanding of the complexity and consequences of the climate crisis, leaving individuals feeling both responsible and powerless. Daily reminders of climate change may act as triggers for distress, highlighting the pervasive nature of this experience. These findings point towards configuring reports on climate change in such a way as to keep CCD manageable as an important putative target of population-based preventive measures. However, the potential long-term impacts of educational and media coverage still need to be elucidated by future research. In addition, findings from this qualitative study offer valuable guidance for mental health promotion and prevention measures for young individuals. Given that most mental health disorders emerge before the age of 25 and the disease burden is highest during adolescence and young adulthood,^30^ measures should take place at an early age. Findings centre on subjective needs for (1) acquiring effective emotion regulation strategies to help individuals manage CCD, (2) supporting individuals in creating opportunities to take meaningful action, (3) strengthening social support and connectedness with like-minded individuals and (4) fostering knowledge about climate change-related emotions, that is, in psychoeducational elements.

## Supplementary material

10.1136/bmjment-2025-301549 online supplemental file 1

## Data Availability

Data are available upon reasonable request.
