# Supplementary material for: Addressing youths’ climate change-related distress: a qualitative study on the experience of burden, triggering and protective factors
Source: BMJ Ment Health. 2025 Oct 2;28(1):e301549. doi: 10.1136/bmjment-2025-301549 (PMC12496112; doi:10.1136/bmjment-2025-301549)
Supplement: online supplemental file 1 [file bmjment-28-1-s001.docx]

Supplementary File 1: Interview Guideline

**Introduction:**

Welcome, and thank you for joining this interview. To start, I’d like to suggest that we address each other informally. Is that okay with you?

[Introduction]

Thank you for taking the time for this interview. We will talk for a maximum of two hours, focusing specifically on the topic of mental health burdens related to climate change. We are particularly interested in your personal experiences, perspectives, and opinions. I hope we can have a relaxed conversation about this.

Everything you share will be treated confidentially. This means we are interested in what you have to say, but not your name or personal information. We will record the conversation so that we can transcribe (i.e., write it down) later. The recording will not be shared with third parties and will be deleted after the analysis is complete. You have already confirmed your consent in the document you signed.

1. **Introduction and Opening Questions (Introducing the Topic)**

- When did you first become aware of the issue of climate change?
- How did you perceive the topic at that time?
- What was your attitude toward this topic back then?
- How has that developed since then? / How has your attitude changed over time?

1. **Understanding the Burden and Exploring Perception in daily life**

[Encourage participants to provide concrete examples throughout this section.]

- How do you feel when you think about climate change? What goes through your mind?

**Follow-up questions:**

- What specific emotions arise when you think about climate change?
  - What about positive emotions?
  - Have you ever felt burdened because of these negative emotions?
  - How did that come to this?
  - How has this burden shown itself in daily life?
  - In what other ways have, you felt burdened?

1. **Identifying Triggers and Understanding the Temporal Course**

- When do your burdens or emotions related to climate change arise in your daily life?
  - In what situations or on what occasions do they occur?
  - Can you identify additional triggers that contribute to these burdens?
- How does the burden progress after a trigger occurs?

[Refer to the triggers already explored.]

- - How long does it take you to return to your baseline after such a trigger?
- If no burdens arise in daily life: Are there specific situations or occasions where the emotions are stronger?
  - Are there specific triggers associated with those emotions?
  - How long does it take after such a trigger for the emotions to return to baseline?

1. **Identifying Protective Factors**

- Are there situations where the burden does not occur or is less intense?
  - What characterizes these situations?
  - Can you think of any other similar situations?
- Are there external factors that help prevent the burden from arising?
- Are there internal factors within yourself that help reduce or prevent the burden in such situations?
- Can you think of any additional internal or external factors that help reduce or prevent the burden?(or make it less intense)

1. **Final Question**

- What else should we know to fully understand your experience with or burden due to climate change?
